# Recognition and Clinical Presentation of Invasive Fungal Disease in Neonates and Children

**DOI:** 10.1093/jpids/pix053

**Published:** 2017-08-31

**Authors:** Jill King, Zoi-Dorothea Pana, Thomas Lehrnbecher, William J Steinbach, Adilia Warris

**Affiliations:** 1 Aberdeen Fungal Group, Medical Research Council Centre for Medical Mycology, Institute of Medical Sciences, University of Aberdeen, and the Royal Aberdeen Children’s Hospital, United Kingdom;; 2 Hospital Epidemiology and Infection Control, Division of Infectious Diseases, Johns Hopkins Hospital, Baltimore, Maryland;; 3 Division of Paediatric Haematology and Oncology, Hospital for Children and Adolescents, Johann Wolfgang Goethe-University, Frankfurt, Germany; and; 4 Division of Pediatric Infectious Diseases, Department of Pediatrics, and Department of Molecular Genetics and Microbiology, Duke University Medical Center, Durham, North Carolina

**Keywords:** clinical presentation, invasive aspergillosis, invasive candidiasis, invasive fungal disease, pediatric patients

## Abstract

Invasive fungal diseases (IFDs) are devastating opportunistic infections that result in significant morbidity and death in a broad range of pediatric patients, particularly those with a compromised immune system. Recognizing them can be difficult, because nonspecific clinical signs and symptoms or isolated fever are frequently the only presenting features. Therefore, a high index of clinical suspicion is necessary in patients at increased risk of IFD, which requires knowledge of the pediatric patient population at risk, additional predisposing factors within this population, and the clinical signs and symptoms of IFD. With this review, we aim to summarize current knowledge regarding the recognition and clinical presentation of IFD in neonates and children.

Invasive fungal disease (IFD) affects primarily patients with a compromised immune system, classically children with a hematological malignancy, especially those with acute leukemia, hematopoietic stem cell transplant (HSCT) recipients, solid-organ transplant (SOT) recipients, those with primary or acquired immunodeficiency, and premature neonates [1–3]. There is also an emerging appreciation of other patient groups at increased risk of IFD, such as those in a pediatric intensive care unit (PICU) [4, 5], patients who suffer a traumatic injury, those who have undergone surgery, particularly abdominal surgery or corrective surgery for congenital heart disease [6, 7], and patients with an autoimmune and/or autoinflammatory condition treated with immunomodulatory agents [8, 9]. Because the clinical disease phenotype is a result of the interaction between a fungal pathogen and the individual host immune response, a variety of disease presentations, determined mainly by the nature of the immune impairment, can be seen [10]. Knowledge of the clinical signs and symptoms of IFD, additional predisposing risk factors, and variations in disease phenotypes across different at-risk patient groups is essential for enabling early recognition and diagnosis of IFD and, ultimately, improving disease outcomes.

## PATIENTS WITH A HEMATOLOGICAL MALIGNANCY AND TRANSPLANT RECIPIENTS

In children with a hematological malignancy or those who have undergone HSCT, persistent febrile neutropenia despite broad-spectrum antibiotic treatment is often the first and only clinical sign to alert the clinician to suspect an IFD [11, 12].

### Risk Factors

An initial risk profile can be derived on the basis of the underlying malignancy or transplant type and the specific characteristics of chemotherapy. For instance, patients with acute myeloid leukemia, high-risk (including relapsed) acute lymphoblastic leukemia, recipients of an allogeneic HSCT (particularly from a matched unrelated donor), and those who suffer from chronic or severe acute graft-versus-host disease are at particularly high risk of infection [1–3, 13–17]. Within the first 30 days after HSCT [14, 18, 19], a period of significant immunosuppression and profound neutropenia, patients are at the greatest risk of IFD. However, a second period of risk exists after neutrophil engraftment that coincides with acute or chronic graft-versus-host disease and requires ongoing vigilance well beyond neutrophil recovery [3, 14, 20]. The risk of developing IFD after autologous HSCT is considerably lower than that after allogeneic HSCT, and underlying disease can affect the incidence of IFD [1, 21, 22].

IFD in children with a malignancy or after HSCT rarely occurs in the presence of an isolated predisposing host factor [17, 23]. Typically, multiple risk factors are present, such as prolonged neutropenia (absolute neutrophil count, ≤500/μL for ≥10 days), high-dose corticosteroid use (≥0.3 mg/kg per day of prednisone or equivalent), additional immunosuppressive therapy or chemotherapy, presence of a central venous catheter (CVC), use of parenteral nutrition, mucositis, concomitant bacterial infection, or preceding broad-spectrum antibiotic use [3, 5, 16, 23–26]. The depth and duration of neutropenia are associated with chemotherapy intensity, and the highest risk for IFD is reported during induction therapy [27, 28]. For instance, during induction chemotherapy to treat acute myeloid leukemia, the risk of invasive candidiasis (IC) is greater than that during subsequent chemotherapy courses (10% vs 6%, respectively) [28]. Data regarding the effect of age as an additional risk factor for IFD in children with a malignancy are conflicting. In 2 studies, an age of >10 years was associated with a higher incidence of IFD [11, 28], whereas a subsequent study found no significant differences between younger and older children [29].

Data concerning risk factors for IFD in pediatric SOT recipients have been more limited. A study that included 1854 pediatric heart transplantation recipients identified previous surgery and mechanical ventilation during transplantation as independent risk factors for IFD in a multivariate analysis [30]. For children who had undergone lung transplantation, colonization before transplantation, transplant rejection, cytomegalovirus mismatch, tacrolimus treatment, and older age increased the risk of developing IFD [31]. In a recently published US study of 397 children who were undergoing liver transplantation, the only significant risk factor for IC identified with multivariate analysis was admission to an ICU before transplantation [32]. Children who undergo a renal transplant are considered to be at low risk for IFD; candidemia related to intravascular catheter use is the most common presentation [33, 34].

### Clinical Presentation

Invasive aspergillosis (IA) and IC are the most common IFDs in patients with a hematological malignancy and in HSCT or SOT recipients [1, 11, 12, 35]. Non-*Aspergillus* molds, such as *Mucorales*, are being seen with increased frequency in patients who have received an *Aspergillus*-active antifungal agent before their IFD is diagnosed [3, 36]. As a result of effective prophylaxis with trimethoprim-sulfamethoxazole, *Pneumocystis jirovecii* pneumonia (PCP) is seen rarely in these patients. However, a diagnosis of PCP should still be considered in patients who receive second-line PCP prophylaxis agents that are likely less effective than trimethoprim-sulfamethoxazole [37].

### Candidiasis

IC is identified most commonly after the isolation of yeasts on blood culture [38, 39]; *Candida* spp now represent the third most common cause of nosocomial bloodstream infection in children [40, 41]. *Candida* spp need to be strongly considered when a clinical diagnosis of sepsis or septic shock in a neutropenic pediatric patient has been made, especially in the presence of an intravascular catheter and recent exposure to broad-spectrum antibiotics. However, although the isolation of *Candida* spp on blood culture is highly specific for IC, the sensitivity of such cultures is low, and disseminated infection can be present in the absence of a positive blood culture result [42–44]. Indeed, approximately half of the patients with IC will not have a positive blood culture result, and it might be more useful to consider IC as 3 distinct clinical entities, that is, candidemia in the absence of deep-seated infection, candidemia with disseminated infection, and deep-seated infection in the absence of candidemia [44, 45]. Dissemination can occur to almost any site, including the lungs, liver, spleen, kidneys, brain, eyes, and heart, although the symptoms of disseminated disease are frequently minimal and might become apparent only on immune reconstitution [45–47]. However, the identification of dissemination is important, because prolonged treatment might be required [45], and it is an independent risk factor for death in children with IC [48].

*Candida* meningitis and meningoencephalitis occur more frequently in children than in adults with candidemia (11.4% vs 0.8%, respectively; *P* < .001) [49] and can present in the absence of candidemia [43]. In keeping with other manifestations of IC, patients are often asymptomatic; 5 (42%) of 12 children with *Candida* meningitis in 1 study displayed no symptoms other than fever [43]. When symptoms were present, the most common presenting feature was a reduced level of consciousness (50%), followed by seizures (33%), headache (25%), nuchal rigidity (25%), and cranial nerve palsies (17%). Skin lesions were also identified in one-third of the patients, which reflects the primarily hematogenous route of infection. Cerebrospinal fluid findings might be unremarkable, and the absence of cerebrospinal fluid abnormalities does not exclude central nervous system (CNS) infection [43].

Ocular involvement during IC is a rare but potentially sight-threatening complication. The most frequent manifestations are chorioretinitis and endophthalmitis [50]. Findings on examination can be unilateral or bilateral, and lesions typically appear as fluffy yellow-white retinal or vitreal balls with associated hemorrhage or vitreous haze [51]. Current practice guidelines from the Infectious Diseases Society of America recommend dilated ophthalmic examination within the first week of candidemia diagnosis in all nonneutropenic patients and within 1 week of neutrophil recovery in all neutropenic patients [45]. Examination findings are often minimal before neutrophil recovery, which explains the recommendation for delayed examination in neutropenic patients [45]. Information on visual outcomes in these patients has been limited; however, in 1 study, 3 of 4 children with endophthalmitis experienced subsequent complications that included retinal detachment and globe rupture [50].

Hepatosplenic candidiasis is rare in children [52], and only sparse information on its clinical presentation is available. Nausea and vomiting, right or left upper-quadrant pain, hepatosplenomegaly, or transaminitis can alert a clinician to the possibility of hepatosplenic infection [53].

Other rare sites of dissemination in children with IC include the heart and skeleton. *Candida* osteomyelitis in children typically affects the femoral metaphysis with complicating septic arthritis in the neighboring joint. The humerus, vertebrae, and ribs are other potential sites of infection. Local symptoms are usually present and include pain, tenderness, overlying erythema and edema, and limitation of movement; a majority of patients are febrile at presentation [54, 55]. Infective endocarditis secondary to *Candida* sp infection can affect children with an underlying hematological malignancy, particularly during periods of immunosuppression, but is seen more commonly in children with preexisting heart disease. Presentation is typically with fever and a new heart murmur. The classical signs of endocarditis, such as Osler’s nodes, Janeway lesions, and Roth spots, are seen rarely in children [56–58].

### Aspergillosis

The primary sites of IA are the lungs and the sinuses, yet only approximately half of the children with pulmonary IA display clinical signs and symptoms of respiratory infection [3, 36, 59–61]. When present, the most commonly reported symptoms are cough, dyspnea, and chest or pleuritic pain [15, 36, 59, 61], and tachypnea and oxygen requirement are the only reported clinical signs [60, 61].

Clinical symptoms of fungal rhinosinusitis can include fever, rhinorrhea, nasal congestion, facial pain or numbness, and headache [60–63]. However, symptoms can be nonspecific, and symptomatic disease might be a late presentation [62]. In a recent study by Cohn et al [60], who used a screening protocol that included direct nasal endoscopy performed at the bedside by an otorhinolaryngologist in addition to computed tomography of the chest and abdominal ultrasound in children with persistent febrile neutropenia despite the administration of broad-spectrum antibiotics, sinonasal disease was confirmed in 13 (42%) of 31 patients. Of the patients identified, 8 (62%) of 13 were asymptomatic, which suggests that fungal rhinosinusitis might be underappreciated in the pediatric oncology population if diagnostic modalities are withheld until specific signs and symptoms occur.

After pulmonary and sinus disease, the most common site of *Aspergillus* infection is the brain [61, 64]; between 6% and 15% of pediatric patients show evidence of CNS infection [17, 36, 59, 61, 65], and CNS symptoms are reported in up to half of all children with disseminated IA [66]. Multiple brain abscesses are the most common radiographic finding, followed by vasculitis and meningoencephalitis, although other more unusual clinical presentations, including intracerebral hemorrhage and hemorrhagic infarcts, have been reported also, which reflects the angioinvasive properties of *Aspergillus* hyphae [66]. Symptoms of brain abscess, such as headache and vomiting, are not typically seen in these patients. Instead, symptoms of disorientation, somnolence, general malaise, focal seizures, hemiparesis, and cranial nerve palsies are associated more frequently with CNS aspergillosis and might be the primary presenting feature of IA [61, 64, 66].

Cutaneous involvement is far more common in children than in adults [17, 61], affecting between 8% and 41% of children with IA in various pediatric studies [17, 23, 36, 59, 61, 67]. This involvement can be a result of either local infection at the site of trauma (such as intravenous cannulas or CVC sites) or hematogenous dissemination. The clinical characteristics of cutaneous lesions vary from ulcers at intravenous sites to macules, papules, and nodular necrotic lesions with or without surrounding erythema and cellulitis [36, 61, 67]. Lesions can appear as purpuric nodules in the extremities as a result of hematogenous dissemination [64] and can progress to form necrotic eschars [67]. Isolated cutaneous *Aspergillus* infection has been associated with a more favorable outcome than those of other manifestations of IA [23, 24, 67, 68]. However, it is important to recognize that cutaneous lesions can represent the first sign of disseminated disease; 2 (33%) of 6 patients with clinically localized disease in a study by Abbasi et al [61] were found to have evidence of disseminated disease at autopsy, and in a more recent study by Burgos et al [17], 9 (47.4%) of 19 patients with cutaneous infection also had infection at other sites. When skin lesions are present, they can provide a useful source of diagnostic specimens; almost one-third of positive culture results in a 10-year retrospective study of invasive mold infections in pediatric oncology patients were isolated from the skin [36].

Cardiac involvement is also rare but has been reported consistently in studies of pediatric IA in neutropenic patients [17, 36, 61]. Clinical presentations include pericardial effusion, intracardiac thrombus, and endocarditis [17, 36, 61]

### Mucormycosis

Similar to aspergillosis, the 2 primary sites of infection for mucormycosis are the pulmonary parenchyma and the sinuses. Among pediatric patients with a malignancy or those who were undergoing HSCT, 1 study found that the main clinical sites of mucormycosis were the lungs (25.6%), skin and soft tissues (12.8%), the paranasal sinuses/sinoorbital region (13.8%), and the rhinocerebral region (9.1%). Disseminated disease was present in 46.5% of these patients, which is higher than with IA [69]. The presenting symptoms of mucormycosis cannot be differentiated easily from those of IA and IFD caused by other molds, although signs and symptoms of hemorrhages and infarction are observed more frequently.

## PATIENTS ADMITTED TO A PICU

Although PICU admission is itself a risk factor for IFD [70], the majority of children who develop IFD in a PICU have other underlying risk factors that precede their PICU admission [71]. These factors include underlying malignancy, immunocompromise, a gastrointestinal disorder (particularly patients with short-gut syndrome), trauma, and surgery (particularly abdominal surgery, neurosurgery, or corrective surgery for congenital heart disease) [5, 71, 72]. In addition, PICU patients often require CVC or urinary catheter placement and frequently receive broad-spectrum antibiotic treatment, parenteral nutrition, and systemic steroid or other immunosuppressive treatment, which further increases their susceptibility to IFD [4, 5, 70, 71, 73].

### Risk Factors

PICU patients are at risk of both IA and IC; however, IC is much more prevalent and has been better characterized [70]. *Candida* spp are now the third most common cause of bloodstream infection and the most frequent cause of IFD in PICU patients [74]. Predictive scoring systems for IC in adult ICU patients exist [75, 76], and attempts were made recently to develop pediatric predictive scoring systems to help identify PICU patients with likely IC [5, 7, 73]. Zaoutis et al [73] were the first to attempt such a system and found that the presence of a CVC or a malignancy or the use of vancomycin or agents with activity against anaerobic organisms for >3 days in the preceding 2 weeks were significant independent risk factors for IC in PICU patients [36]. The authors developed a prediction model by combining the aforementioned factors (>10% risk) and observed a predicted probability of candidemia that ranged from 10.7% to 46% [73]. However, an attempt to validate the prediction model in a multicenter study proved unsuccessful [77]. A separate predictive IC probability model for PICU patients, the ERICAP scoring system, was developed by Jordan et al [5]. It assigns points for the following clinical factors, identified in a multivariate analysis as significantly increasing the likelihood of IC in PICU patients and demonstrating high specificity for IC when present in combination: a pre-PICU hospital stay of ≥15 days; fever; thrombocytopenia; and use of parenteral nutrition [5]. Motta et al [7] also proposed a predictive scoring system for candidemia in children after surgery for congenital heart disease. They found the combination of a RACHS-1 (Risk Adjustment for Congenital Heart Surgery) (a scoring system that groups cardiac procedures into 1 of 6 categories on the basis of risk of death) score of ≥3, thrombocytopenia, and use of acid-suppression therapy resulted in a 58% predictive probability of candidemia. However, both scoring systems remain to be validated.

Another study found significant species-specific differences in risk factors; in particular, IC attributed to *Candida albicans* was associated significantly with chronic metabolic disease, gastrointestinal surgery, fever at PICU admission, and parenteral nutrition, whereas *Candida parapsilosis–*specific risk factors were previous yeast colonization, tracheostomy, parenteral nutrition, thrombocytopenia at PICU admission, and previous bacterial infection [5, 72]. Species-specific (*C albicans* versus non-*albicans Candida* spp) risk factors for IC in PICU patients were identified also in a study by Hegazi et al [78]; they found that the risk factors for acquiring non-*albicans* IC were an age of >1 year and isolation of a *Candida* species from a CVC or endotracheal tube. Further investigation to validate and improve the proposed clinical prediction models are of utmost importance to enable clinicians to better identify children in the PICU who are at the highest risk for IC and could benefit from targeted prophylactic or preemptive antifungal treatment.

### Clinical Presentation

Identifying IFD in PICU patients can be exceedingly difficult because symptoms frequently are indistinguishable from sepsis secondary to bacterial infection, and fever refractory to antibiotic treatment is the most common presenting feature [5, 72]. It is unfortunate that no study has addressed the clinical presentation of IFD in pediatric PICU patients, aside from the specific populations highlighted in this review. However, the presence of thrombocytopenia often raises the concern of IC in PICU patients. Recent studies associated pronounced and prolonged thrombocytopenia with candidemia in PICU patients after corrective surgery for congenital heart disease [5, 7] and in premature neonates [79–81]. Further investigation and validation of thrombocytopenia as a heralding sign of IC is warranted.

## PREMATURE NEONATES

Neonates possess a number of endogenous and exogenous risk factors that predispose them to IFD, caused mainly by *Candida* spp [82, 83]. Few of these factors are intrinsic but instead have been associated with the immaturity of the immune system in these patients [83–85].

### Risk Factors

Immaturity of the premature neonate’s epidermis and intestinal mucosal barriers enable *Candida* spp to translocate from the skin or gastrointestinal tract into the bloodstream [86]. Birth weight is correlated inversely with the incidence of IC; incidences range from 4% to 16% in extremely-low-birth-weight infants and 2% to 5% in very-low-birth-weight infants [87–90]. Apart from their immunosuppressed status, premature infants are often exposed to several risk factors for IC inherent in the provision of prolonged intensive care. In particular, these risk factors include parenteral nutrition, mechanical ventilation, central venous access, proton pump–inhibiting agents, postnatal corticosteroid use, and broad-spectrum antibiotic exposure (particularly to third-generation cephalosporins and carbapenems). Intestinal pathology and abdominal surgery, both common among neonatal intensive care unit patients, have been identified as risk factors also [87, 88, 91–96].

Colonization with *Candida* spp before the onset of IC is common among neonates; colonization rates range from 18% to 26% [84, 97–99]. Sources of colonization vary by *Candida* spp. For instance, *C parapsilosis* colonization occurs via horizontal transmission, typically >7 days after neonatal intensive care unit admission, whereas *C albicans* colonization occurs via vertical transmission in the perinatal period [100]. A recent study conducted by Barton et al [101] found that chorioamnionitis and vaginal delivery were strongly associated with the development of early-onset candidiasis (at ≤ 7 days of life).

Although *Candida* spp are the predominant source of IFD in premature neonates, other fungal pathogens are sometimes opportunistic in this patient population. *Malassezia* spp are a group of lipid-dependent yeasts that frequently colonize the skin and gastrointestinal tract but are an infrequent cause of neonatal fungemia [102]. Infection with *Malassezia furfur* has been associated with the use of lipid infusions via a CVC in neonates [103]. IFD of the skin and soft tissues caused by molds such as *Aspergillus* spp and *Mucorales* have been reported infrequently. These infections have been described in relation to mild local trauma and skin contamination from the use of wooden tongue depressors, arm boards, and/or adhesive tapes [104–106].

### Clinical Presentation

The most common presentation of IFD in premature neonates is a generalized sepsis that is indistinguishable from late-onset bacterial sepsis [107]. In a majority of premature infants with an IFD, infection presents around the third week of life and is caused predominantly by *Candida* spp. Almost 25% of candidemia infections in premature neonates are associated with a meningoencephalitis, even when no overt neurological symptoms are present [108]. Dissemination to the kidney (5%), eye (3%), and heart (5%) is seen, particularly in neonates with persistent candidemia [109, 110]. Isolated infections in the CNS [111], kidneys [112], heart [113], and bones and joints [114] in the presence of indwelling devices have been reported. *Candida* infection of the kidneys in neonates can be complicated by the development of a fungal bezoar (fungal ball) and lead to urinary tract obstruction [115]. Neonates with IC develop thrombocytopenia more frequently than those with bacteremia and have both a lower platelet nadir and a longer duration of thrombocytopenia [79–81]. In combination with the aforementioned existing risk factors, candidemia should be suspected in a neonate with clinical signs of sepsis and new thrombocytopenia. Hyperglycemia is also a common feature of neonatal fungal sepsis and acts as a clinical predictor of IC; the odds of IC increase as the blood glucose level rises [87].

Infections caused by *Aspergillus* spp and *Mucorales* in neonates are often localized to the skin and soft tissues and affect the most premature and extremely low birth weight neonates, who have an impaired and immature skin barrier function [104–106]. Clear differences in the common sites of mucormycosis can be seen between premature neonates and older children with a malignancy. Gastrointestinal (54%) and cutaneous (36%) diseases are the predominant phenotypes in neonates, whereas sinopulmonary and rhinocerebral patterns of disease are noted mainly in older children with a malignancy [116].

## PRIMARY AND ACQUIRED IMMUNODEFICIENCY

IFD is highly unusual in the absence of impaired immunity but can represent the primary presenting feature of an underlying immunodeficiency. Therefore, the identification of IFD in an otherwise healthy child should prompt further investigation into possible immunodeficiency, and investigations should be targeted toward the most probable defective arm of host defense.

### Risk Factors

The main risk factor for IFD in children with immunodeficiency is the underlying immunodeficiency itself, and specific deficiencies in the host defense place the patient at risk for specific fungal pathogens [117]. Classical examples of this include IA with chronic granulomatous disease (CGD) and PCP in patients with severe combined immunodeficiency and acquired immunodeficiency secondary to human immunodeficiency virus (HIV) [118–123].

Deficiencies in T-cell immunity are the main predisposing factor for PCP, and patients at increased risk of infection include those with severe combined immunodeficiency, HIV, CD40 ligand deficiency, nuclear factor κB (NF-κB) essential modulator (NEMO) deficiency, hyperimmunoglobulin E syndrome (hyper-IgE) (Job syndrome), and X-linked hyperimmunoglobulin M syndrome [124, 125]. Prophylaxis with cotrimoxazole is highly effective, and breakthrough infection during prophylaxis should prompt consideration of noncompliance or antimicrobial resistance [37].

The majority of cryptococcosis cases occur in children with defective cell-mediated immunity, caused mainly by HIV infection or a primary immunodeficiency such as hyperimmunoglobulin M syndrome, hyper-IgE syndrome, and GATA2 deficiency [126, 127].

Disorders of host phagocyte function, such as CGD, place patients at increased risk for invasive mold infections such as those caused by *Aspergillus* spp and *Mucorales*. Indeed, patients with CGD remain at the highest lifetime risk of IA, despite effective antifungal prophylaxis [128–131]. Infections caused by *Mucorales* are rare and typically are seen in the setting of immunosuppressive treatment for inflammatory complications of CGD [132]. After infancy, IC is relatively uncommon in patients with CGD. However, the presence of additional risk factors, such as prolonged antibiotic treatment and the use of CVCs, places patients with CGD at increased risk for candidemia [131, 133].

Although primary immunodeficiencies with impairment of interleukin 17 (IL-17) immunity traditionally present with chronic mucocutaneous candidiasis and have not been considered to confer increased risk of IFD [134], there are case reports of IC in such patients, including *Candida* endocarditis in a child with hyper-IgE syndrome [135] and meningoencephalitis due to *Candida* in children and adults with previously unrecognized CARD9 deficiency [136–138]. Therefore, consideration should be given to the possibility of an underlying IL-17 immunodeficiency, such as STAT3 or CARD9 deficiency, in a previously healthy child who presents with disseminated candidiasis.

### Clinical Presentation

Classical PCP presents with hypoxia in excess of the degree of respiratory distress in a young infant (typically 3–6 months old). In contrast to most causes of pneumonia in infants, pyrexia and preceding coryzal symptoms frequently are absent. Instead, these children typically have a history of progressive dyspnea and dry cough with low-grade pyrexia, tachypnea, and hypoxia found on examination but an absence of adventitious sounds on auscultation [124, 139, 140]. Respiratory distress often progresses rapidly and necessitates significant respiratory support [124, 141].

Cryptococcosis most commonly manifests as meningoencephalitis, disseminated disease, and pneumonia. Pulmonary cryptococcosis without dissemination is a recognized but unusual clinical presentation in immunocompromised children [142–144]. The most common presenting clinical symptoms and signs in a review that included 53 pediatric patients were headache (79%), fever (77%), vomiting (70%), and neck pain and/or nuchal stiffness (49%). Hydrocephalus was reported for 6 patients [145]. It is notable that 26% of the children in this Brazilian study had no predisposing condition, and only 25% of the children had an underlying diagnosis of acquired immunodeficiency syndrome. The average age of the children was 7.7 years (range, 0–16 years), and only 5.6% were <2 years of age [145]. Other studies have found a comparable age distribution, with cryptococcosis occurring more frequently in middle childhood (6–12 years) and rarely appearing during the first 2 years of life [146, 147]. The most recent series of pediatric cryptococcosis came from a national survey of Colombian children <16 years of age [148]. Twenty-four percent of the children were HIV positive, and their mean age was 8.4 years, which is comparable with that in previously published studies [145–147]. In the Colombian series, neurocryptococcosis (87.8%) was most common, followed by disseminated disease (12.2%). Among the 5 patients with disseminated disease, 2 had skin involvement. Clinical signs and symptoms and their frequencies were similar to those reported from a study by Severo et al [145]. Results of several studies have suggested that disseminated cryptococcal disease is rare [145, 147, 148], although disseminated cryptococcosis was diagnosed in 47.8% of 23 pediatric patients in a case series in China. The affected organs in those 11 children included the lungs (n = 11), CNS (n = 7), lymph nodes (n = 10), liver (n = 9), and spleen (n = 7) [144]. None of the children had an identified immunocompromising condition, but 7 of them were malnourished. The differences in underlying disorders and geographical aspects might explain the variation observed in the clinical entities of cryptococcal disease.

Failure to thrive was the most common (71%) presenting feature of IFD in children with CGD registered in the French National Database for Primary Immunodeficiency over a 25-year period [2]. The clinical presentation of IA in patients with CGD can be highly variable but is often indolent with minimal symptoms [118, 149]. Fever is reported in 61% in those presenting with IA [119]. However, a review of a French cohort of patients with CGD found that 37% reported neither fever nor respiratory symptoms at the time of IFD diagnosis [150], which is comparable with the one-third of patients with IA who presented to the National Institutes of Health who were asymptomatic at the time of diagnosis [151]. When present, symptoms associated with invasive pulmonary aspergillosis can include chest discomfort, cough (usually nonproductive), and progressive dyspnea, but hemoptysis is rare [119]. The second most common site of IA in patients with CGD is the bones, often with multifocal lesions; the thoracic vertebrae and ribs are affected most commonly [152, 153]. Clinical features have not been well described, but the most common manifestations seem to be localized pain and tenderness, often without fever. Vertebral invasion is associated with signs of spinal cord invasion in 45% of cases. Localized brain abscesses caused by *Aspergillus* spp are considered rare [133, 154, 155]. Clinical features vary from mild fever and headaches to seizures and localizing signs that mimic space-occupying lesions [119]. Other less common sites of *Aspergillus* infection include skin, lymph nodes, liver, and spleen [119, 133]. The clinical manifestations of skin infections are diverse, from erythematous plaques and papules to pustules and purulent ulcers, localized mainly to the extremities. Hepatic and splenic abscesses caused by infection with an *Aspergillus* sp typically are seen during disseminated infection rather than occurring in isolation [119].

*Candida* spp are the most common cause of fungal meningitis, fungemia, and fungal lymphadenitis in patients with CGD [119]. Young infants with CGD (ranging from 8 weeks to 4 months old) seem to be more prone to developing IFD caused by a *Candida* sp. Clinical signs are those of a septic infant (fever and irritability) sometimes associated with local signs of organ involvement, such as lymphadenopathy and hepatosplenomegaly, or signs of CNS involvement.

## SUMMARY

A wide variety of vulnerable pediatric patients are at risk of developing IFD as a consequence of either an intrinsic impairment of the immune system, an acquired disorder, intensive management that interferes with normal immune function, or direct immunosuppressive therapies. Because the clinical manifestations of IFD are a result of the interaction between a fungus and the host immune system, a variety of disease phenotypes can be recognized. Nevertheless, clinical signs and symptoms are often nonspecific and develop late during disease progression. A high a priori clinical suspicion is needed and should be based on the severity and characteristics of the immune dysfunction and the presence of additional risk factors. Several features separate pediatric IFD from those that occur in adults, and these pediatric-specific features should be taken into account when developing clinical guidelines for and definitions of IFD. The European Organization for Research and Treatment of Cancer/Invasive Fungal Infections Cooperative Group and the National Institute of Allergy and Infectious Diseases Mycoses Study Group (EORTC/MSG) are currently preparing a second update of the consensus definitions of IFD [156] in which pediatric-specific features that aid in disease recognition are taken into account. This important step forward will increase the applicability of the consensus definitions in pediatric populations and facilitate the identification of IFD for clinical, epidemiological, and research purposes.

## Notes

***Financial support.*** Prof Warris and Dr King are supported by the Wellcome Trust Strategic Award (grant 097377) and the Medical Research Council Centre for Medical Mycology (grant MR/N006364/1) at the University of Aberdeen.

***Supplement sponsorship.*** This article appears as part of the supplement “State of the Art Diagnosis of Pediatric Invasive Fungal Disease: Recommendations From the Joint European Organization for the Treatment of Cancer/Mycoses Study Group (EORTC/MSG) Pediatric Committee,” sponsored by Astellas.

***Potential conflicts of interest.*** All authors: No reported conflicts of interest. All authors have submitted the ICMJE Form for Disclosure of Potential Conflicts of Interest. Conflicts that the editors consider relevant to the content of the manuscript have been disclosed.
